# Comparing the Efficacy and Safety of Apixaban Versus Warfarin in Morbidly Obese Patients

**DOI:** 10.7759/cureus.30303

**Published:** 2022-10-14

**Authors:** Sultan N Alotaibi, Hani Hasan, Hend Metwali, Mohammed Aseeri

**Affiliations:** 1 Pharmaceutical Care Services, King Abdulaziz Medical City, Jeddah, SAU; 2 College of Medicine, King Saud Bin Abdulaziz University for Health Sciences, Jeddah, SAU; 3 Pharmaceutical Care Services, King Abdulaziz Medical City Jeddah, Jeddah, SAU

**Keywords:** bleeding, venous thromboembolism, morbid obesity, warfarin, apixaban

## Abstract

This study was conducted to evaluate the efficacy and safety of apixaban versus warfarin in morbidly obese patients. A total of 250 morbidly obese patients with a body mass index (BMI) higher than 40 kg/m^2^ or a body weight higher than 120 kg who were on anticoagulation therapy with either apixaban or warfarin for over one month were included in the study. This retrospective cohort, multicenter study was executed using the medical records of 125 morbidly obese patients treated with apixaban, while patients on warfarin were selected using a systemic random sampling to match the sample size of the apixaban group. There was no significant difference between apixaban and warfarin in the development of thromboembolic events and major bleeding. However, incidences of minor bleeding significantly decreased in the apixaban group compared to patients treated with warfarin. This difference was overcome by controlling serum creatinine and nonsteroidal anti-inflammatory drugs (NSAIDs). In conclusion, apixaban efficacy and safety are nearly the same as that of warfarin in morbidly obese patients with a lower incidence of minor bleeding with apixaban. Controlling serum creatinine and NSAIDs use may improve warfarin safety and decrease its complications.

## Introduction

Direct oral anticoagulants (DOACs), including apixaban, edoxaban, rivaroxaban, and dabigatran, were approved by the Food and Drug Administration (FDA) in 2010 as alternative oral anticoagulation therapies to warfarin [1]. They offer benefits over standard-of-care warfarin for the treatment of venous thromboembolism (VTE) or for stroke prevention in non-valvular atrial fibrillation (NVAF) patients because there is no need for routine laboratory monitoring and dietary restrictions and because of the reduced incidence of drug-drug interactions [2].

The FDA approved apixaban for preventing stroke and systemic embolism in patients with non-valvular atrial fibrillation (AF) in 2012. In August 2014, its use was expanded to the treatment of deep vein thrombosis (DVT), pulmonary embolism (PE), and thromboprophylaxis in patients undergoing hip or knee replacement surgery [3]. The volume of distribution of apixaban is approximately 21 liters and may vary based on the patient’s weight. The oral bioavailability of apixaban is 50%, 27% of which is cleared by the kidney as an unchanged drug [4]. It is a direct oral anticoagulant agent that selectively and reversibly inhibits free fraction and clot-bound factor Xa. Apixaban dosing is highly variable based on the indication [5].

Obesity is a serious condition and cause of significant morbidity and mortality. It increases the risk of type 2 diabetes, recurrent thromboembolism, AF, and acute coronary syndrome [6]. It is anticipated that by 2030, obese people will constitute more than half the general population and more than 10% will have morbid obesity (BMI > 40) [7]. The available data about the efficacy and safety of apixaban in morbidly obese patients is limited. Also, few clinical trials have been carried out to assess its efficacy and safety in morbidly obese patients in comparison to warfarin [8]. Interestingly, apixaban has been inappropriately prescribed, dispensed, and administered to morbidly obese patients who otherwise would not have been ideal candidates to receive apixaban [9]. Recently published studies found that high serum creatinine levels and inappropriate use of NSAIDs may significantly modulate the efficacy and safety of apixaban in obese patients [10-11]. Thus, the present study was carried out to evaluate the efficacy and safety of apixaban versus warfarin in patients with a BMI higher than 40 or a body weight higher than 120 kg. This was assessed by evaluating the rate of major thromboembolic events, such as stroke, transient ischemic attacks (TIA), systemic embolism, DVT, or PE in morbidly obese patients who were treated with apixaban or warfarin. In addition, the rate of bleeding events was evaluated in both groups.

## Materials and methods

This study was carried out at King Abdul-Aziz Medical City in the central and western regions of Saudi Arabia and included surgical patients and those in the intensive care unit. It is a tertiary care hospital with 750 beds. The study was approved by the institutional review board at the Ministry of National Guard-Health Affairs (MNGHA: study no. RJ19/055/J). The study is a retrospective cohort, multi-center study executed using the medical records of 125 morbidly obese patients who were treated with apixaban. Patients on warfarin were selected using a systemic random sampling method to match the sample size of the apixaban group. Their data were pulled from the Information Services Division department, which has patients’ medical record numbers, medication names, doses, frequencies, initiation dates, discontinuation dates, heights, weights, and BMIs. The study included patients older than 18 years with a BMI above 40 kg/m^2^ or body weight higher than 120 kg who were on anticoagulation therapy with apixaban or warfarin for more than one month, starting from March 2016 (the date apixaban was added to the formulary) till March 2019. These patients were identified from the hospital information system (BESTCare2.0A). Pregnant patients with indications for anticoagulation other than AF or VTE, patients who received apixaban or warfarin for less than one month before the study, and who were shifted from warfarin to apixaban or vice versa were excluded from this study.

AF and VTE were diagnosed based on the documented history of these conditions in the medical record. Individual patients’ data included BMI, warfarin, DOAC or NSAID use, ischemic stroke events, transient ischemic attack (TIA), major bleeding, minor bleeding, life-threatening bleeding events, serum creatinine, and other clinical demographics. Baseline characteristic data were selected from the most recent values prior to the initial dose of anticoagulation therapy. Efficacy and safety outcome events were recorded if the event was documented in the medical record of the healthcare system.

The hemoglobin (Hgb) level was recorded from the first day of medication administration and was considered the baseline level. All Hgb values were considered during the treatment period until the third day after discontinuation of the medications to compare the baseline with the lowest Hgb readings. The bleeding severity was classified into major bleeding, minor bleeding, and life-threatening bleeding. Major bleeding was defined as a reduction in the Hgb level to at least 2 g/dL below the Hgb baseline, transfusion of at least two units of blood, or symptomatic bleeding in a critical area or organ (intracranial, intra-abdominal, intra-spinal, retro-peritoneal, intra-articular, or deep muscle hematoma). Life-threatening bleeding was defined as fatal bleeding, bleeding with a decrease in the Hgb level of at least 5 g/dL below the Hgb baseline, or bleeding requiring transfusion of at least four units of blood or inotropic agents or necessitating surgery. Minor bleeding was defined as any bleeding other than major or life-threatening bleeding [12].

The Shapiro-Wilk normality test was used to check data normality before proceeding to the statistical comparisons. The values were analyzed using Statistical Package for Social Sciences (SPSS) software version 26 (IBM Corp., Armonk, NY) and the results were presented as mean ± standard deviation (SD) and median (25-75 IQR) or number (%) for parametric and nonparametric parameters, respectively. The significance of the categorized data and the parametric data were assessed using Pearson’s chi-square test and the Mann-Whitney test, respectively. Multiple logistic regression analyses were performed to examine the relationship between apixaban or warfarin use and serum creatinine level, NSAID use, thromboembolic manifestations, and minor and major bleeding. A p-value less than 0.05 was regarded as the minimally accepted significance level.

## Results

In the present study, we used data of 250 morbidly obese patients who had been on either apixaban (n = 125) or warfarin (n = 125) therapy for more than one month. The mean age of the patients in the apixaban group was 67.68 ± 11.53 years, while the mean age was 60.66 ± 14.54 for the warfarin group (p = 0.0001). In the apixaban group, 36.8% of the patients were below 65 years old, while 60.8% were below 65 years old in the warfarin group (p = 0.0001). Regarding participants’ gender, males represented 15.8% of the apixaban group and 20.8% of the warfarin group. Regarding body weight, the mean for the apixaban group was 109.95 ± 18.57 kg and 111.79 ± 19.17 kg for the warfarin group (p = 0.485). The mean value for BMI was 45.82 ± 7.19 for patients in apixaban therapy and 45.28 ± 6.26 for patients in warfarin therapy (p = 0.925). Duration of treatment in the apixaban group was 20.38 ± 11.71 months and 21.51 ± 12.45 months in the warfarin group (p = 0.303). In addition, 15.2% of patients on apixaban had a history of stroke, while 13.6% of patients on warfarin did. However, the history of stroke was unknown in 16.8% of patients treated with apixaban and 6.4% of patients on warfarin (p = 0.028). Among the patients on apixaban, 85.6% had AF and 14.4% had VTE vs 52.8% and 47.2%, respectively, of patients on warfarin, (p = 0.0001). Aspirin was administered to 14.4% of patients on apixaban and 12% of patients on warfarin (p = 0.355), clopidogrel to 11.2% of patients on apixaban and 3.2% to patients on warfarin (p = 0.013), NSAIDs to 20.8% of patients on apixaban and to 19.2% of patients on warfarin (p = 0.437), and steroids were prescribed to 20.8% of patients on apixaban and to 17.6% of patients on warfarin (p = 0.315; Table 1).

**Table 1:** Demographic characteristics of the patients* Abbreviations: IQR: Interquartile range, SD: Standard deviation, BMI: Body mass index, NSAIDs: Non-Steroidal Anti-Inflammatory Drugs. *Data were expressed as mean+/-SD, median (25-75 IQR), or number (%) as appropriate. Significance between categorized data was made using the Person Chi-Square test and between parametric data using the Mann-Whitney test.

Characteristics	Apixaban (n= 125)	Warfarin (n= 125)	P -value
Duration of treatments (Months), median (IQR)	20.00 (12.00-31.5)	22.00 (9.0-35.0)	0.303
Age (years), mean±SD	67.68±11.53	60.66±14.54	0.0001
Age category			0.0001
< 65 years	46 (36.8%)	76 (60.8%)	
≥ 65 years	79 (63.2%)	49 (39.2%)	
Gender			0.259
Male	21 (15.8%)	26 (20.8%)	
Female	104 (83.2%)	99 (79.2%)	
Weight (kg), mean±SD	109.95±18.57,	111.79±19.17	0.485
BMI (kg/m^2^), mean±SD	45.82±7.19,	45.28±6.26	0.925
History of stroke			0.028
No	85 (68.0%)	100 (80.0%)	
Yes	19 (15.2%)	17 (13.6%)	
Unknown	21 (16.8%)	8 (6.4%)	
Diagnosis			0.0001
Atrial fibrillation	107 (85.6%)	66 (52.8%)	
Venous thromboembolism	18 (14.4%)	59 (47.2%)	
Medications used			
Aspirin	18 (14.4%)	15 (12.0%)	0.355
Clopidogrel	14 (11.2%)	4 (3.2%)	0.013
NSAIDs	26 (20.8%)	24 (19.2%)	0.437
Steroids	26 (20.8%)	22 (17.6%)	0.315

Table 2 shows the complications encountered during the apixaban therapy and the warfarin therapy. It shows that 5.6% of patients in the apixaban group and 7.2% of those in the warfarin group (p = 0.608) reported an incidence of major thromboembolic manifestations. Major bleeding occurred in 5.6% of patients on apixaban and 4.8% of patients on warfarin (p = 0.5), while there was minor bleeding in 4% of patients on apixaban and 14.4% of those on warfarin (p = 0.004).

**Table 2:** Complications of treatments*. Abbreviation: TIA: Transient ischaemic attack, DVT: Deep vein thrombosis, PE: Pulmonary Embolism. *Data were expressed as number (%). Significance between categorized data was made using the Person Chi-Square test.

Characteristics	Apixaban (n= 125)	Warfarin (n= 125)	P -value
Major thromboembolic manifestations	7 (5.6%)	9 (7.2%)	0.608
Stroke	5 (4.0%)	3 (2.4%)	0.361
TIA	-	1 (0.8%)	0.500
DVT	2 (1.6%)	1 (0.8%)	0.500
PE	-	4 (3.2%)	0.061
Major bleeding	7 (5.6%)	6 (4.8%)	0.500
Minor bleeding	5 (4.0%)	18 (14.4%)	0.004

There was no significant difference in the impact of apixaban and warfarin use on thromboembolic, major, or minor bleeding after controlling for serum creatinine and NSAIDs use in comparison to the pre-control values (p = 0.505). The serum creatinine control values did not affect the incidence of major bleeding in either group when compared to the pre-control values (p = 0.134). However, the incidence of minor bleeding was significantly affected by the control of serum creatinine levels when compared with the pre-control values (p = 0.288 post-control vs 0.004 pre-control; Table 3). Control of NSAID use did not significantly affect the incidence of thromboembolic manifestations or major bleeding between the apixaban and warfarin groups when compared to the pre-control values (p = 0.899 and 0.699 respectively). Furthermore, the incidence of minor bleeding was significantly affected by the control of NSAID use when compared to the pre-control values (p = 0.772 post-control vs 0.004 pre-control; Table 3).

**Table 3:** Impact of apixaban or Warfarin Use on Thromboembolic, major and minor bleedings after controlling serum creatinine and NSAID use. Abbreviation: NSAIDs: Non-Steroidal Anti-Inflammatory Drugs, CI: Confidence interval. Multivariate logistic regression analysis. Reference: Normal creatinine and no use of NSAID.

Variable	Odd’s ratio	95% CI	P value
Thromboembolic complications			
Creatinine serum levels	0.585	0.121-2.833	0.505
NSAID	0.918	0.245-3.435	0.899
Major bleeding			
Creatinine serum levels	0.344	0.085-1.391	0.134
NSAID	1.357	0.288-6.396	0.699
Minor bleeding			
Creatinine serum levels	0.522	0.157-1.731	0.288
NSAID	1.182	0.381-3.672	0.772

## Discussion

Obesity is a health problem that affects an increasing number of the population worldwide [13]. Recent studies have shown that there is a high incidence of stroke, AF, and VTE in a wide proportion of morbidly obese patients [14]. This was attributed to the increase in intra-abdominal pressure, which exerts a mechanical strain on the abdominal and pelvic vessels, together with the associated hypercoagulable state generated by the increased serum levels of transforming growth factor-beta 1 (TGF-β1) and pro-inflammatory cytokines [15]. In addition, morbid obesity is predisposed to increased levels of the von Willebrand factor, prothrombin, factor VII, factor VIII, and fibrinogen, resulting in an increased incidence of thromboembolic events [16]. Moreover, Vyas and Lambiase suggested that the hemodynamic changes associated with high BMI may alter cardiac structure and physiology, increasing susceptibility to the development of AF [17]. Ghattas et al. reported that adipocytokines and growth factors generated by excess adipose tissue may diffuse into the myocardium, resulting in local inflammation and myocardial fibrosis, which have arrhythmogenic effects [18]. This result was in agreement with the findings of the present study in which 69.2% of the participants had a history of AF, 30.8% had a history of VTE, and 14.4% had a history of stroke.

Furthermore, no significant difference was found between patients on apixaban and those on warfarin regarding the incidence of thromboembolic manifestations. This finding is similar to the results of the following studies. Yun et al., who conducted a retrospective cohort study comparing the incidence of clinical outcomes (VTE recurrence, stroke, and bleeding) in morbidly obese patients treated with apixaban or warfarin, found similar rates between cerebrovascular accident (CVA) incidence in patients with NVAF (p = 0.56), and no statistical difference in VTE recurrence rates (p = 0.77) [19]. However, they found that the bleeding rate was lower with apixaban than with warfarin (P = 0.03). Kido and Ngorsuraches assessed the efficacy and safety of DOAC (rivaroxaban, dabigatran, and apixaban) in patients with AF or flutter in morbidly obese patients at a tertiary care hospital. They found that patients who received DOACs showed no statistically significant difference in the incidence of stroke or TIA compared with those on warfarin. However, the patients who received apixaban did not have any incidence of stroke or TIA [20].

In the present study, there was no significant difference between patients on apixaban and those on warfarin regarding incidences of major bleeding (p = 0.5). This finding is in line with Elshafei et al., who conducted a meta-analysis study that included five observational studies of 6,585 patients. They found that DOACs, including apixaban, produced no significant risk of major bleeding compared to warfarin [21].

Contrary to our results, Cohen et al. reported that apixaban-using morbidly obese patients had a significantly lower risk of recurrent VTE and major bleeding compared to warfarin. Their study included 43,095 obese and morbidly obese patients, and they found that apixaban is associated with a significantly lower risk of VTE (obese: 0.73 {0.64-0.84}; morbidly obese: 0.65 {0.53-0.80}) and major bleeding (obese: 0.73 {0.62-0.85}; morbidly obese: 0.68 {0.54-0.86}) in comparison to patients on warfarin therapy [22].

In our study, patients on apixaban recorded a significant decrease in the incidence of minor bleeding compared to patients on warfarin (p = 0.004). These findings were in accordance with Kjerpeseth et al. who identified treatment-naïve patients, including morbidly obese patients, who initiated warfarin, dabigatran, rivaroxaban, or apixaban for NVAF. After follow-up for one year, all DOACs were just as effective as warfarin in the prevention of ischemic stroke, TIA, or systemic embolism. Safety from bleeding was similar or better, including less intracranial bleeding with all DOACs, less gastrointestinal bleeding with apixaban, and fewer other types of bleeding in patients using dabigatran or apixaban compared to those taking warfarin [23].

Renal functions were proven to significantly modulate the efficacy and safety of oral anticoagulants [24]. DOACs were reported to have altered pharmacokinetic properties in patients with renal dysfunction, but limited data are available regarding their use in these patients [25]. Apixaban proved to have the greatest number of therapeutic outcomes in patients with renal dysfunction, supporting its use over warfarin in this population [26]. This concurs with the results of the present study where control of serum creatinine levels induced a significant decrease in incidences of minor bleeding in warfarin-treated patients compared to the pre-control values, reaching a level similar to the incidence of minor bleeding in patients treated with apixaban.

The prolonged use of NSAIDs was reported to significantly affect the pharmacokinetic and pharmacodynamic properties of oral anticoagulants [27]. Kent et al. conducted a study on 2,279 patients who used NSAIDs and either DOAC or warfarin during the trial. With the use of NSAIDs, the incidence of stroke, VTE, or bleeding significantly increased in patients on warfarin therapy. The rate of hospitalization was higher in patients on warfarin compared to patients treated with a DOAC [11]. Another systematic review and meta-analysis study was published by Villa Zapata et al. who concluded that the incidence of bleeding was significantly elevated in patients taking warfarin and an NSAID or COX-2 inhibitor together when compared to patients taking warfarin alone [28]. This is in agreement with our findings in which the control of NSAID use was associated with a significant decrease in the incidence of minor bleeding in the warfarin group compared to the pre-control values, approximating the incidence of minor bleeding in patients treated with apixaban.

## Conclusions

Apixaban efficacy and safety were nearly the same as that of warfarin in morbidly obese patients. Apixaban led to less development of minor bleeding compared to warfarin in this population. Control of serum creatinine and NSAID use may significantly improve the safety of warfarin and decrease the complications of its prolonged use. These results might add valuable evidence to the available limited data and call for further research about the use of apixaban in morbidly obese patients.
